# Modeling aortic diseases using induced pluripotent stem cells

**DOI:** 10.1002/sctm.20-0322

**Published:** 2020-11-12

**Authors:** Kai Zhu, Wenrui Ma, Jun Li, Yu Shrike Zhang, Weijia Zhang, Hao Lai, Chunsheng Wang

**Affiliations:** ^1^ Department of Cardiac Surgery, Zhongshan Hospital Fudan University Shanghai People's Republic of China; ^2^ Shanghai Institute of Cardiovascular Diseases Shanghai People's Republic of China; ^3^ Division of Engineering in Medicine, Department of Medicine, Brigham and Women's Hospital Harvard Medical School Cambridge Massachusetts USA; ^4^ Institutes of Biomedical Sciences and Department of Systems Biology for Medicine, Shanghai Medical College Fudan University Shanghai People's Republic of China; ^5^ The State Key Laboratory of Molecular Engineering of Polymers Fudan University Shanghai People's Republic of China

**Keywords:** aortic disease, disease modeling, in vitro, induced pluripotent stem cells, precision medicine

## Abstract

Induced pluripotent stem cells (iPSCs) offer an effective platform for studies of human physiology and have revealed new possibilities for disease modeling at the cellular level. These cells also have the potential to be leveraged in the practice of precision medicine, including personalized drug testing. Aortic diseases result in significant morbidity and mortality and pose a global burden to healthcare. Their pathogenesis is mostly associated with functional alterations of vascular components, such as endothelial cells and vascular smooth muscle cells. Drugs that have been proven to be effective in animal models often fail to protect patients from adverse aortic events in clinical studies, provoking researchers to develop reliable in vitro models using human cells. In this review, we summarize the patient iPSC‐derived aortic cells that have been utilized to model aortic diseases in vitro. In advanced models, hemodynamic factors, such as blood flow‐induced shear stress and cyclic strain, have been added to the systems to replicate cellular microenvironments in the aortic wall. Examples of the utility of such factors in modeling various aortopathies, such as Marfan syndrome, Loeys‐Dietz syndrome, and bicuspid aortic valve‐related aortopathy, are also described. Overall, the iPSC‐based in vitro cell models have shown the potential to promote the development and practice of precision medicine in the treatment of aortic diseases.

## Graphical abstract

Application of iPSCs to establish in vitro models for research of aortic diseases.

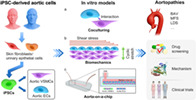

**Significance statement**

Aortic diseases carry significant morbidity and mortality and pose a worldwide burden to healthcare, for which few drugs have been shown effective in clinical trials. Patient‐derived induced pluripotent stem cells, when being integrated into in vitro models mimicking aortic microenvironment and biomechanics, can serve as a useful tool for more precise drug selection and mechanism investigation. This article summarizes previous in vitro models in the research of aortic diseases and provides guidance for further development of advanced models using induced pluripotent stem cells.

## 1 INTRODUCTION

Aortic diseases pose a heavy healthcare burden due to the significant mortality and morbidity.^1^ A wide spectrum of diseases involve the aorta, including aortic aneurysm (AA), aortic dissection (AD), atherosclerotic diseases, infection, and traumatic injuries. AA, which results from the progressive dilation of the aortic wall, is the most common type of aortic disease and may lead to lethal outcomes of AD or aortic rupture.^2^ Despite advances in surgical treatment in recent years, perioperative mortalities after open and endovascular interventions for AD (2.6%‐39%) remain high.^3^, ^4^ Drugs that have been proven effective in animal models often fail to protect human subjects from adverse aortic events in clinical studies.^5^ Additionally, there is a lack of reliable methods to predict the risk of AD or rupture in patients with chronic AA, partly because of the complicated etiologies of aortic disease and the mechanisms that are yet to be elucidated.

To address these problems, the development of disease models is necessary to improve our understanding of the underlying pathophysiology of aortic diseases and predict responses to medications.^5^ Traditionally, animal models have been extensively used in investigations of human diseases. However, due to the myriad etiologies of human aortic diseases, it is both technically difficult and costly to construct pathologically relevant models in animals. Moreover, animal models are less reliable in drug selection because of species differences and are flawed in terms of controlling for hemodynamic confounders. in vitro primary cell‐based models, which usually include cells extracted from donor human aortae, can more directly reflect human aortic pathophysiology than animal models. Unfortunately, donor aorta sources are extremely limited, and aortic primary cells cannot be conveniently harvested to evaluate the individual responses to therapeutic agents.^6^

Over the past decade, induced pluripotent stem cells (iPSCs) have revolutionized the field of biomedical research, and they represent a useful research tool for precision medicine.^6^, ^7^ Individual somatic cells can be reprogrammed into iPSCs by transfection of a set of four transcription factors (Oct4/Sox2/c‐Myc/Klf4). iPSCs can subsequently be differentiated into a wide variety of functional somatic cells. As an alternative cell model, iPSCs circumvent many of the problems associated with animal and primary cell models and allow for expansion of patient‐specific functional somatic cells.^8^ Moreover, iPSC‐based disease models may allow individualized evaluations of responses to certain treatment schemes, an approach known as precision medicine.^8^ Here, we review the components of the aortic wall, the differentiation of iPSCs into aortic cells, and efforts toward replicating the aortic wall microenvironment in iPSC models in vitro. Additionally, we discuss how iPSC‐based platforms are being used in modeling several aortic diseases.

## 2 MICROSTRUCTURES OF THE AORTIC WALL

**FIGURE 1:**
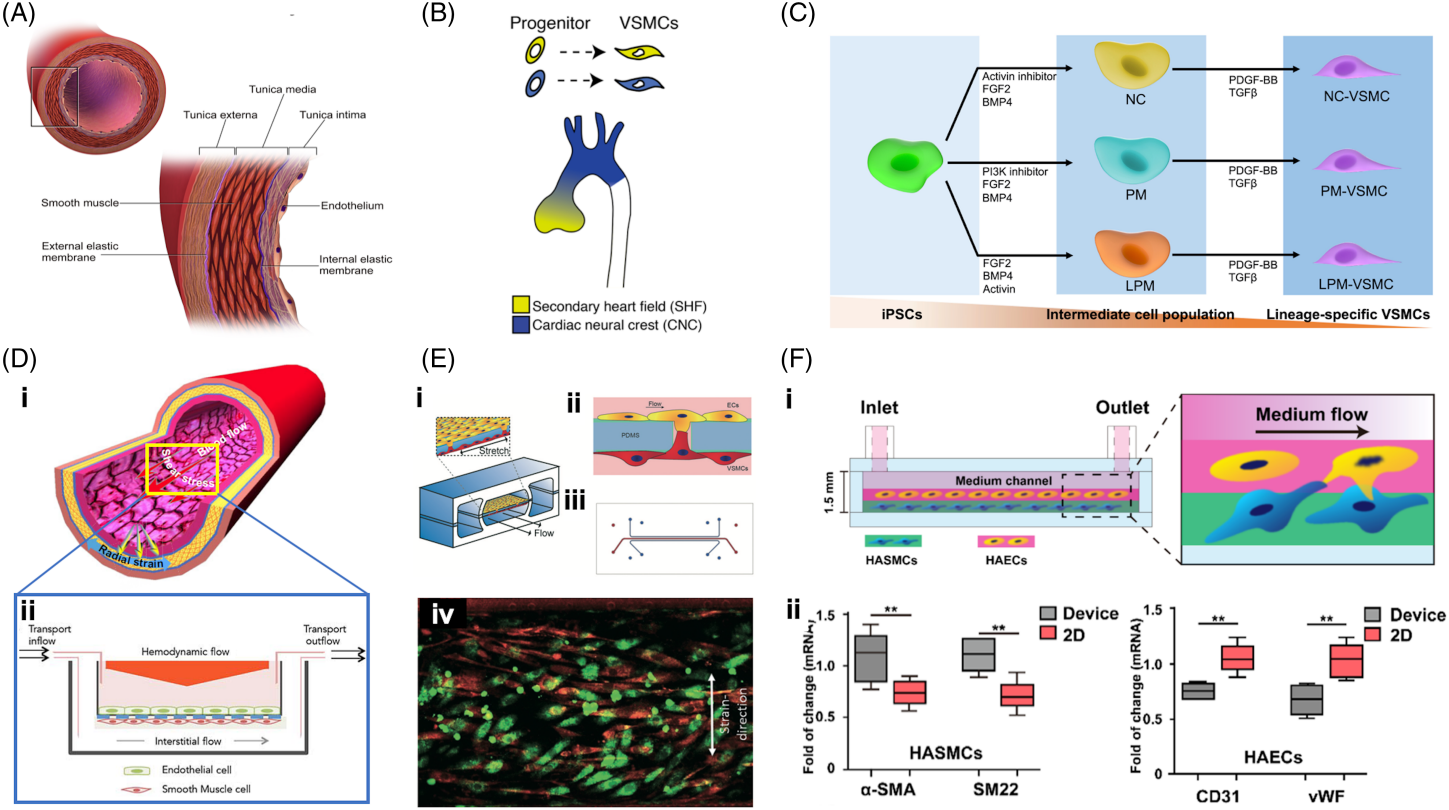
Aortic microstructures, cells, and in vitro models. A, Cellular and matrix components of the human aorta (reprinted with permission, Blausen.com^9^). B, In the ascending thoracic aorta, the aortic VSMCs are derived from two cell lineages, the secondary heart field and the neural crest (reprinted with permission, MacFarlane et al^10^). C, Accordingly, VSMCs from distinct lineages can be differentiated from human iPSCs with different combinations of agents. D, (i) The aortic wall is exposed to blood flow‐induced shear stress, cyclic strain, and hydrostatic pressure (reprinted with permission, Dan et al^11^); and (ii) an in vitro coculturing model mimicking blood and interstitial flows within the aorta (reprinted with permission, Collado et al^12^). E, (i‐iii) In a vessel‐on‐a‐chip model, VSMCs and ECs were cocultured on a porous, tensile membrane, which underwent cyclic strain and flow shear stress, and (iv) in response to these biomechanical stimuli, VSMCs could align perpendicularly to the strain direction and ECs exhibited a cobblestone‐like morphology (reprinted with permission, van Engeland et al^13^). F, (i) Another example of an aorta‐on‐a‐chip model made using a combination of bioprinting technology. VSMCs and ECs were mixed with the bioinks separately and bioprinted into a microfluidic device; and (ii) compared with isolated 2D‐cultured cells, cocultured VSMCs exhibited an increase in the contractile phenotype, and the expressions of CD31 and von Willebrand factor were downregulated in ECs (reprinted with permission, Abudupataer et al^14^)

All aortic diseases are associated with changes in the microstructures of the aortic wall, which is a highly organized tissue consisting of vascular cells and extracellular matrix (ECM). The aortic wall can be divided into three distinct layers known as the intima, adventitia, and media (Figure 1A).^9^, ^15^, ^16^ The intima is composed of a single layer of endothelial cells (ECs) attached to the basal lamina. The adventitia consists of fibroblasts, fibrotic tissues, and vasa vasorum. The media, which is in between the intima and adventitia, comprises approximately 80% of the wall thickness and contains elastin lamellae, collagen, microfibers, and vascular smooth muscle cells (VSMCs). As the most abundant ECM components in the media, elastin and collagen fibers, which are mainly synthesized and deposited by VSMCs, are responsible for aortic mechanical strength. Therefore, to keep the aortic microstructures intact, a balance between ECM and VSMCs must be subtly maintained. In pathological situations, VSMC dysfunction may offset this balance and result in aortic diseases. For instance, VSMCs may undergo phenotypic switching from the contractile to the synthetic type, which is characterized by increased collagen synthesis and matrix metalloproteinase (MMP)‐2 production that lead to increased collagen deposition and elastin degradation, decreased structural strength of the aortic wall, and increased susceptibility to AA.^17^ In addition, in models of aortic diseases, the maladaptive interaction between fibroblasts and VSMCs is also reported to affect the structural and functional integrity of the aorta via promotion of stress‐related signaling in VSMCs and activation of fibroblasts.^18^

The aortic wall is also a nonuniform structure with distinct sections, each of which is more susceptible to certain types of disease than other sections.^10^ Embryologists have proven that the aortic cells may have different embryonic origins, and the population of VSMCs comprising the aortic microstructures is consequently heterogeneous. In the ascending aorta and aortic arch, VSMCs are derived from the neural crest; VSMCs in the descending thoracic aorta are derived from the paraxial mesoderm, while neural crest‐ and secondary heart field‐derived VSMCs intermingle in the aortic root (Figure 1B).^10^ The heterogeneity of VSMCs leads to section‐specific microphysiology in the aortic wall and differences in the vulnerability of VSMCs to pathogenic stimuli. For instance, bicuspid aortic valve (BAV)‐related aortopathy usually occurs in the proximal thoracic aorta and is rarely found in the descending thoracic aorta.^10^, ^19^ Therefore, VSMCs derived from different iPSC lineages may enable us to develop a more detailed understanding of section‐specific aortic diseases, thereby optimizing decision‐making in clinical diagnosis and treatment.

## 3 HUMAN iPSCs‐DERIVED AORTIC ECs AND SMCs

### 3.1 Human iPSCs‐derived ECs

To obtain iPSCs‐derived ECs, induction of iPSCs into mesodermal progenitors is first required, using a combination of factors including activin A, bone morphogenetic protein‐4 (BMP4), and basic fibroblast growth factor (FGF). EC specification is subsequently triggered by the addition of vascular endothelial growth factor (VEGF). In addition, GSK3 inhibition and transforming growth factor‐β (TGF‐β) inhibition are also established methods that promote the differentiation of iPSCs into mesodermal lineages cells and ECs.^20^ The differentiation efficiency of ECs usually ranges from 10% to 35% of the differentiated cell population. Magnetic‐activated cell sorting is utilized to purify the differentiated ECs, which are characterized by the formation of tube‐like networks, upregulation of surface adhesion molecules in response to pro‐inflammatory molecules, nitric oxide (NO) production, and uptake of acetylated low‐density lipoprotein. To obtain ECs with vessel‐specific characteristics, small molecules and growth factors are added to modify cellular signaling. Studies have found that high levels of VEGF‐A and NOTCH signaling could be key drivers of arterial specification.^21^, ^22^ In EC specification of mesoderm progenitors, VEGF‐A can be utilized to promote arterial EC differentiation. With the addition of an inositol monophosphatase inhibitor and NOTCH agonist, the artery‐like iPSC‐ECs exhibited increased NO production, reduced leukocyte adhesion, and improved responses to shear stress; moreover, the expression of venous markers, such as NR2F2, was obviously downregulated.^21^ Moreover, vascular bed‐specific hemodynamic and mechanical factors should also be considered, as biomechanical cues are likely required for the differentiation of arterial human iPSC‐ECs. Studies have demonstrated that exposure of human iPSC‐ECs to a high, arterial‐like shear stress (1‐2 Pa) could further promote the differentiation of arterial ECs.^23^

### 3.2 Human iPSCs‐derived VSMCs

During embryonic development, VSMCs originate from different mesoderm lineages, including the lateral plate mesoderm, neural crest, paraxial mesoderm, secondary heart field, and proepicardial organ. They are subsequently recruited to the endothelial tubes from the surrounding tissues and further mature in line with their functions in their specific location in the vascular bed. Therefore, VSMCs present different characteristics in different vascular tissues and even in distinct sections of the aorta.^10^ Accordingly, many protocols have been investigated to induce the differentiation of different subtypes of VSMCs from different mesoderm lineages (Figure 1C).^24^ For example, to obtain lateral plate mesoderm, a combination of BMP4, FGF2, and activin can be utilized. Inducing the differentiation of neural crest mesoderm requires a combination of FGF2 and an activin inhibitor. For the differentiation of paraxial mesoderm, BMP4, FGF2, and phosphatidylinositol‐4,5‐bisphosphate 3‐kinase (PI3K) inhibitor are required. Subsequently, supplementation with platelet‐derived growth factor‐BB (PDGF‐BB) and TGF‐β is performed to induce the differentiation of VSMCs. Different lineage‐specific VSMCs can be distinguished according to their phenotypic markers. Further maturation of iPSC‐derived VSMCs may be achieved by administration of tensile strains in a cyclic uni‐ or bi‐axial fashion, which benefit cell alignment, increase elastin deposition, and improve calcium signaling and contraction.^25^

## 4 MODELING THE MICROPHYSIOLOGY OF THE AORTIC WALL

Various in vitro models have been reported in the literature that have emulated the pathophysiological features of aortic diseases more effectively than traditional two‐dimensional (2D) cultures. However, most of these models have used pooled VSMC/EC lines or primary cells expanded from patients' aortic tissues, which do not readily allow individualized drug testing for personalized medicine. iPSC‐derived VSMCs/ECs, on the other hand, can be noninvasively harvested and readily integrated into these models. In this regard, we believe that a proper review of these in vitro aortic models is necessary to broaden the implementation of iPSCs in modeling aortic diseases.

### 4.1 Coculturing of cells to model cellular crosstalk

The coculturing system is an in vitro model widely utilized to recapitulate intercellular relationships. Coculturing of iPSC‐derived ECs and VSMCs could enhance cellular crosstalk in a biomimetic microenvironment and promote their maturation of arterial characteristics. Tan et al developed a robust bottom‐up approach to construct open‐ended and tubular three‐dimensional (3D) EC/VSMC coculturing constructs that emulated the human vascular morphology.^23^ Compared with traditional 2D planar coculturing, 3D coculturing takes into account the specific dimensionality and surface curvatures, thus representing more physiologically relevant cell growth patterns. Similarly, in a comparison study of single cell culture and coculturing models, the influence of shear stress on VSMCs was shown to be quite different between the two types of models, and EC‐VSMC interactions could significantly promote VSMC differentiation.^26^, ^27^ Chiu et al developed an in vitro model in which ECs and VSMCs were cocultured on two sides of a chamber separated by a porous membrane.^26^ The surface of the ECs was subjected to an adjustable flow. It was found that VSMC modulation of EC expression of adhesion molecules under flow conditions was mediated via close interactions between VSMCs and ECs. Shear stress could act as a protective regulator of pathology‐related gene expression in vascular ECs cocultured with VSMCs. Furthermore, adventitial fibroblasts were reported to have maladaptive paracrine interactions with VSMCs in aortic disease models.^18^ In the future, it could be helpful to explore new therapeutic targets for treatment of aortic diseases using models comprising cocultured fibroblasts and other vascular cells.

### 4.2 Use of biomechanical stimuli to model hemodynamics

Hemodynamic factors, such as blood flow‐induced shear stress and cyclic strain, act on the aortic wall and generate biomechanical stimuli (Figure 1D).^11^ These hemodynamic factors have been integrated into in vitro models for mechanistic investigations. Abnormal hemodynamics have been shown to affect the phenotypes, proliferation, and migration of VSMCs and consequently to result in functional alterations.

Cyclic stretch is an important hemodynamic factor that influences VSMC functions. It arises from the pulsatile change in the vascular circumference with blood flow. Physiological or pathological cyclic stretch can be integrated into in vitro cell models to investigate the disease mechanisms of VSMCs. VSMCs sense cyclic stretch and transduce it into intracellular signals, resulting in alterations in related gene expression and cellular behaviors, such as proliferation, migration, phenotypic transition, apoptosis, and ECM remodeling.^28^

Shear stress can regulate the release of secretory molecules, such as NO, prostacyclin (PGI2), PDGF‐BB, TGF‐β, and microRNAs (miRs). The uptake of NO, PGI2, and exosome‐embedded miR‐143/miR‐145 by VSMCs leads to functional targeting and the promotion of a quiescent phenotype.^29^ In contrast, release of PDGF‐BB, TGF‐β1, and argonaute 2 (Ago2) in complex with miR‐126 by ECs subjected to low or oscillatory shear stress induced the activation of VSMCs.^29^ Shear stress regulates VSMC functions by a mechanism involving MMP‐2 and PDGF through the NO signaling pathway.^28^, ^29^ In in vitro VSMC models exposed to regulatable laminar shear stress, VSMC proliferation was inhibited by high shear stress and promoted by low shear stress. Recently, Collado et al developed a hemodynamic coculture system and demonstrated that coculturing recapitulated the responses of VSMCs to pathological stimuli, such as those observed in vascular diseases (Figure 1D).^12^

### 4.3 Use of the organ‐on‐a‐chip to model microphysiology

Over the past decade, models using organ‐on‐a‐chip technology have been developed to allow in vitro studies of the physiopathology of various diseases. Generally, organ‐on‐a‐chip devices are based on microfluidic systems featuring submillimeter culture chambers, which can be connected to each other via semipermeable barriers or microchannels. Culture medium flows in the culture chambers at controlled rates. Cells are seeded within the culture chambers in a 2D or 3D fashion, and can be stretched, compressed, or twisted as observed in a physiological or pathological state in vivo, providing more accurate emulation of microphysiology than conventional on‐plate culture systems. In recent years, novel organ‐on‐a‐chip platforms that contain multiple cell types have become an attractive choice to recapitulate physiology and pathology at the organ level.^30^

Organ‐on‐a‐chip technology has also been utilized to investigate interactions between ECs and VSMCs in aortic diseases. Typically, the majority of VSMCs cultured on static, stiff, and 2D surfaces exhibit a synthetic phenotype, while cells on a 3D matrix or under cyclic stretch conditions have a contractile phenotype.^31^ One recent study developed a microfluidic device that enabled the coculturing of interacting ECs and VSMCs under hemodynamic conditions.^13^ In this model, the vascular cells remained viable during prolonged culture, exhibited physiological morphology and organization, and made intercellular contacts. During dynamic culturing of the device in the presence of shear stress of 1 to 1.5 Pa and a strain of 5% to 8%, VSMCs aligned perpendicularly to the strain direction, and ECs adopted a cobblestone morphology (Figure 1E). Another work reported a modified 3D system for coculturing ECs and VSMCs using bioprinting technology, which allowed effective and controllable organization of the cocultured cells.^14^ In response to cyclic stretch, ECs and VSMCs exhibited reduced expression of CD31 and von Willebrand factor and increased expression of ACTA2 and SM22, respectively. It was speculated that the cyclic tensile strains resulted in paracrine signaling in ECs that could be transmitted to VSMCs to produce further functional modulations (Figure 1F). Overall, cocultured organ‐on‐a‐chip models have become a promising tool to study the intercellular behaviors under physiological conditions.

## 5 USE OF iPSCs FOR MODELING AORTIC DISEASES

### 5.1 Marfan syndrome aortopathy

Marfan syndrome (MFS) is a heritable disorder of connective tissue commonly characterized by skeletal abnormalities, aortic dilatation, and ectopic lentis. Large vessels are usually affected by thoracic AA, AD, or rupture at an early age. MFS is caused by a mutation in the FBN1 gene encoding the ECM protein fibrillin‐1. Concurrently, surgery is the only definitive treatment and is recommended for MFS patients once the maximal diameter of the thoracic AA reaches 45 mm.^32^ On the other hand, early management and precision medicine are under investigation for use in delaying the progression of thoracic AA and reducing the risk of catastrophic AD or rupture.

In vitro MFS patient‐specific cell models have served as an effective tool in multiple studies of pathogenesis, drug screening, and gene‐editing therapy.^33^, ^34^, ^35^ Recent studies also utilized iPSC‐derived aortic cells to investigate the pathogenesis of MFS aortopathy. Granata et al developed an iPSC‐VSMC stretch model to recapitulate the pathology of and determine drug responses in MFS aortas.^36^ The iPSCs were derived from dermal fibroblasts of MFS patients and induced to differentiate into three mesodermal cell lineages. Among them, neural crest‐derived VSMCs emulating those of the ascending aorta underwent physiological cyclic stretching. Both the canonical and noncanonical TGF‐β signaling pathways were hyperactive in the model, which was consistent with the immunohistochemical results. In addition, three different drugs (losartan, doxycycline, and a TGF‐β inhibitor) were tested in this model. Among them, losartan was the most effective drug in reducing ECM degradation; however, losartan showed only a partial rescue effect for the impairment of cell proliferation and no effect on cell death. Furthermore, the mutation in the FBN1 gene was corrected by the CRISPR‐Cas9 editing technology, and the corrected MFS‐iPSC line exhibited similar levels of expression of genes involved in the TGF‐β signaling pathway. Based on these findings, it is reasonable that iPSCs would provide an optimal platform for investigations of pathogenetic mechanisms, identification of novel therapeutic targets, and optimization of gene therapies. Clinical studies might be considered to validate the in vivo responses of MFS patients to certain drugs if positive results are shown in the models.

### 5.2 BAV‐related aortopathy

BAV is the most common congenital cardiovascular malformation, with an incidence of 0.9% to 2%.^37^ BAV is frequently associated with proximal AA or AD, which is known as BAV‐related aortopathy and occurs in approximately 20% to 84% of BAV patients, resulting in a 4‐fold elevated risk of thoracic AA compared with that in patients with normal tricuspid aortic valves.^37^ Moreover, only ≤1% of BAV patients with and without aortopathy are affected by mutations in various genes, such as NOTCH1, SMAD6, and ROBO4, which impede universal genetic imputation, establishment of animal models, and the discovery of effective medications.^38^ To date, there has been no drug that has been validated to prevent or retard the development of BAV‐related aortopathy.

Efforts have been made to emulate BAV‐related aortopathy in in vitro models. Jiao et al obtained iPSCs from healthy volunteers and knocked out the NOTCH1 gene.^39^ The NOTCH1^−/−^ (NOTCH1 homozygous knockout) iPSCs and isogenic control iPSCs were induced to differentiate into neural crest mesoderm lineage cells, which subsequently differentiated into VSMCs. Consequently, it was found that VSMCs derived from NOTCH1^−/−^ iPSCs exhibited decreased expression of contractile proteins. Although this was the first reported BAV‐related aortopathy cell model, it should be noted that iPSCs derived from patients with different gene mutations are required to provide comprehensive knowledge of the disease.^40^ Additionally, almost all BAV‐related aortopathies involve the proximal aorta only, which could be partly attributed to the different embryonic origins of VSMCs. To explore the underlying mechanism, different mesoderm lineage‐derived VSMCs were investigated and compared in an in vitro model. In another study, iPSCs from BAV patients were induced to differentiate into neural crest stem cell‐derived VSMCs.^41^ These cells exhibited significantly decreased expression of the VSMC differentiation marker MYH11 and impaired contraction compared with the normal control VSMCs. In contrast, paraxial mesoderm cell‐derived VSMCs were similar to control cells in these aspects. Neural crest stem cell‐derived VSMCs from BAV/thoracic AA patients also showed decreased TGF‐β signaling based on the phosphorylation of SMAD2, and an increase in mTOR signaling. Inhibition of the mTOR pathway using rapamycin rescued the aberrant differentiation of cells.

### 5.3 Loeys‐Dietz syndrome aortopathy

Loeys‐Dietz syndrome (LDS) is an autosomal dominant heritable disease caused by mutations in TGFBR1/2 or SMAD2/3, all of which encode the key components involved in TGF‐β signaling. Although LDS presents phenotypic characteristics similar to those of MFS in large vessels, the skeleton and skin, the aortopathy in LDS is prone to rupture at a smaller size, predisposing LDS patients to a greater risk of aorta‐related mortality if the aortopathy is not identified or treated at an early stage. Several iPSC lines that have been developed from cells from LDS patients with relevant gene mutations.^42^, ^43^ In future studies, iPSC‐derived ECs and VSMCs can be integrated into in vitro models, which may serve as a useful tool in the exploration of pathological mechanisms of LDS and drug selection for treating LDS.

### 5.4 Other hereditary aortopathies

There are other rare hereditary diseases that are associated with an increased risk of aortic diseases, such as Williams‐Beuren syndrome, Turner syndrome, Ehlers‐Danlos syndrome (type IV), familial thoracic aneurysm and dissection, and arterial tortuosity syndrome. It is difficult to develop effective treatments for these rare vascular diseases due to their small affected population bases. Therefore, iPSC‐based models offer major opportunities to identify potential therapeutic targets. Williams‐Beuren syndrome (WBS) involves an elastin gene mutation, and the most common associated lesion is supravalvar aortic stenosis, which has been modeled using iPSCs in a 2D culture system (Supporting Information Table).^44^, ^45^ Using models, it was found that iPSC‐derived VSMCs from WBS patients demonstrated an immature proliferative phenotype with reduced functional and contractile properties, and rapamycin was able to rescue the phenotype. In the future, iPSCs derived from patients with these rare aortic diseases could also be used to construct in vitro models and assess potential therapeutic drug candidates.

## 6 LIMITATIONS AND PERSPECTIVES

**FIGURE 2:**
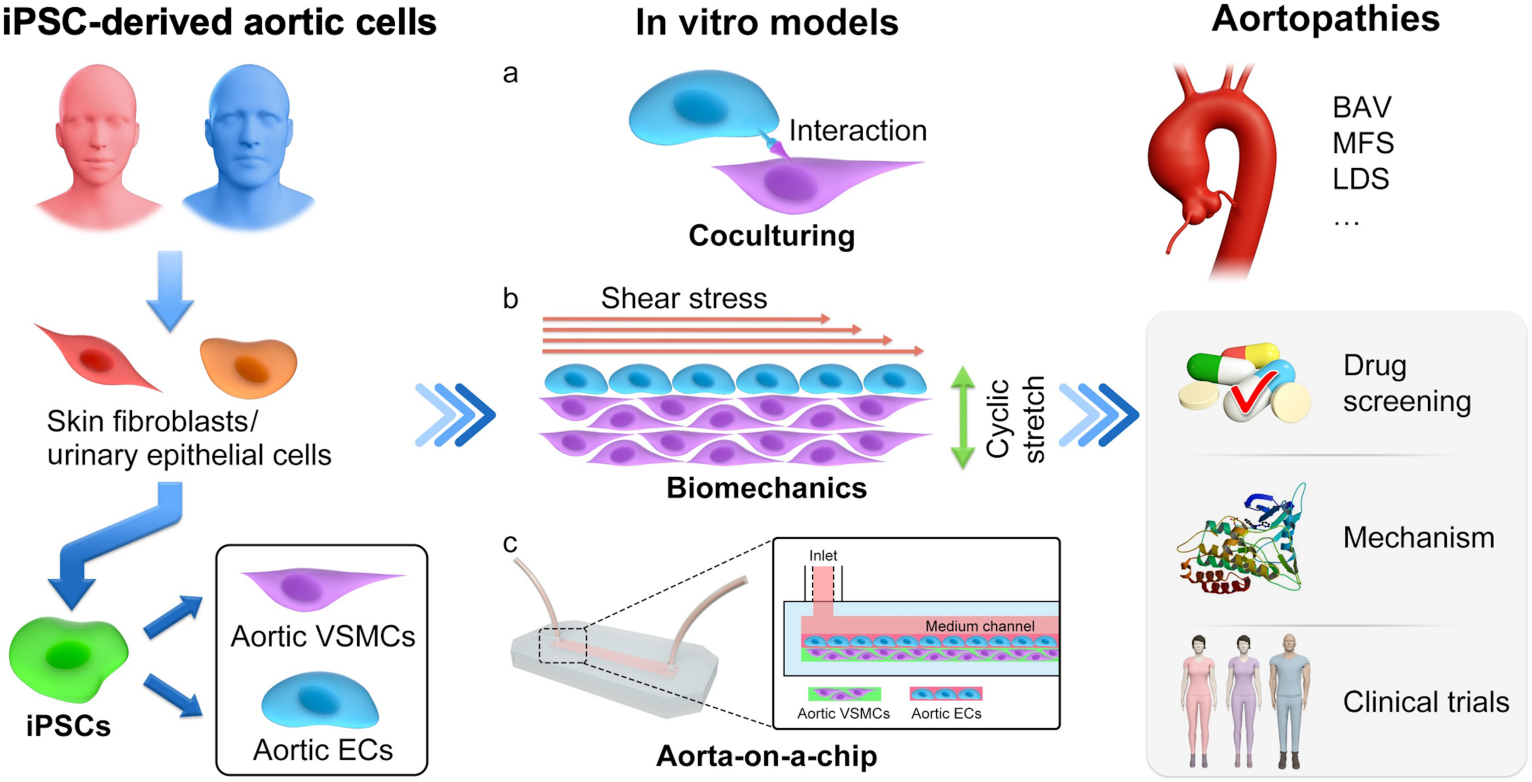
Applications of iPSCs to establish in vitro models for studies of aortic diseases. Using minimally invasive techniques, somatic cells can be harvested from patients with aortic diseases and induced to differentiate into iPSCs. Subsequently, aortic cells of different lineages are differentiated, screened, and included in in vitro models. After integrating the biomechanical factors, aortic disease‐related models are established that recapitulate the pathophysiological characteristics of the aortic tissues. Further studies, namely, drug selection and mechanism dissection studies, can be readily performed in the validated models, the results of which should provide important insights into drug development and clinical trials

In vitro models based on relevant human iPSC‐derived cell types are a promising tool for precision medicine in aortic diseases. Nevertheless, barriers remain to be crossed before their widespread applications in mechanism dissection and the pharmaceutical industry (Figure 2). First, to enhance their clinical utility, the iPSCs used in the models should undergo rigorous validation to guarantee their differentiation into cells that faithfully recapitulate the hallmarks of diseased cells and tissues.^46^ It has been reported that iPSCs are prone to differentiate into somatic cells with an immature identity instead of a completely adult state, which reduces the validity of the disease models. Engraftment and biomechanical stimulation may promote iPSC maturation.^46^ Another issue is the genetic heterogeneity of iPSCs. Previous studies have identified significant genetic variations in iPSCs derived from seemingly “normal” individuals, which may generate baseline fluctuations that affect the analyses of the models generated from these cells. Alternatively, patient‐derived iPSCs with genetic correction of disease‐causing mutations may serve as isogenic controls in disease modeling studies.^46^ Furthermore, iPSC‐derived ECs and VSMCs are useful sources for vascular tissue engineering as grafts.^47^ In aortic diseases caused by specific gene mutations, the genetic correction of iPSCs could also be used to efficiently construct vascular grafts that may repair or replace the diseased vasculature.

Regarding the design and realization of iPSC‐based models, one of the challenges is determining how to accurately emulate disease‐related biomechanical stimuli. More realistic emulation of stimuli relies on accurate and reproducible measurements of the biomechanics of normal and diseased aortas. Furthermore, more sophisticated organ‐on‐a‐chip models that integrate 3D cocultured iPSC‐derived cells and biomechanical factors are warranted. Advanced 3D biofabrication strategies, such as bioprinting, can be applied for model construction.^48^, ^49^, ^50^ Notwithstanding these challenges, recent advances have made the use of iPSCs an irreplaceable tool in in vitro disease models, facilitating the discovery of novel and precise therapeutics for aortic diseases in addition to enhancing the understanding of the fundamental pathological mechanisms.

## CONFLICT OF INTEREST

Y.S.Z. declared consultant/advisory role with Allevi Inc and research funding from NIH, NSF, AHA (not for this research), BRI (for this research). The other authors declared no potential conflicts of interest.

## AUTHOR CONTRIBUTIONS

K.Z.: conception and design, collection and/or assembly of data, financial support, manuscript writing; W.M., J.L., Y.S.Z., W.Z.: collection and/or assembly of data, manuscript writing; H.L., C.W.: conception and design, final approval of manuscript.

## Supporting information


**Data S1**: Supporting Information

## Data Availability

Data sharing is not applicable to this article as no new data were created or analyzed in this study.
