# Supplementary material for: Modeling aortic diseases using induced pluripotent stem cells
Source: Stem Cells Transl Med. 2020 Nov 12;10(2):190–7. doi: 10.1002/sctm.20-0322 (PMC7848399; doi:10.1002/sctm.20-0322)
Supplement: Supplementary file 1 — Data S1: Supporting Information [file SCT3-10-190-s001.docx]

**Supplemental Table.** A brief summary of iPSC-based aortic disease models in literature.

| **Disease** | **Gene mutation** | **iPSC differentiation** | **Model** | **Gene editing** | **Drug treatment** | **Reference** |
| --- | --- | --- | --- | --- | --- | --- |
| MFS | FBN1 | Neural crest VSMCs | 2D stretch model (1 Hz, 10% strain, 24 h; static 20% strain) | CRISPR-mediated FBN1 | Anti-TGF-β antibody; losartan; PD98059 (MAPK kinase inhibitor); SB203580 (MAPK kinase inhibitor) | 35 |
| BAV | NOTCH1 (homozygous knockout by CRISPR/Cas9) | Neural crest VSMCs; Cardiovascular progenitor cell-derived ECs | 2D static model | - | - | 38 |
|  | Unknown | Neural crest VSMCs | 2D static model | - | Rapamycin | 40 |
| WBS | ELN | VSMCs | 2D static model | - | U0126 (MAPK kinase inhibitor) | 43 |
|  | ELN | VSMCs | 2D static model | - | Synthetic elastin-binding protein ligand 2; rapamycin | 44 |

BAV, bicuspid aortic valve; CRISPR, clustered regularly interspaced short palindromic repeat; ECs, endothelial cells; MFS, Marfan Syndrome; VSMC, vascular smooth muscle cells; WBS, Williams-Beuren Syndrome.
